# Supplementary material for: Metabolomic Profiling of Adults with Congenital Heart Disease
Source: Metabolites. 2021 Aug 9;11(8):525. doi: 10.3390/metabo11080525 (PMC8398700; doi:10.3390/metabo11080525)
Supplement: Supplementary file 1 [file metabolites-11-00525-s001.zip › metabolites-1314029-supplementary.pdf]

Table S1. Final multivariable regression models assessing the association between principal components loads, individual metabolites and adverse cardiac characteristics and outcomes.

**O_2 saturation < 95%**					
Variable	*Rel*	*OR*	*LCL*	*UCL*	*p-value*
Single ventricle	.	16.36	4.40	60.75	<0.001
GCDCA	98.4%	1.33	0.99	1.79	0.06
Serotonin/tryptophan	64.4%	0.86	0.78	0.94	0.002
ADMA/Arg	95.8%	1.32	1.03	1.68	0.03
Betaine	61.8%	1.06	1.01	1.11	0.02
**Cardiac Dysfunction**					
Variable	*Rel*	*OR*	*LCL*	*UCL*	*p-value*
PC2	77.4%	0.89	0.82	0.97	0.01
PC5	61.4%	1.15	1.02	1.29	0.02
Citrulline	89.2%	1.09	1.02	1.16	0.02
Cystine	65.6%	1.01	1.00	1.03	0.04
1-Methylhistidine	60.2%	1.15	1.02	1.29	0.02
**RV Dysfunction**					
Variable	*Rel*	*OR*	*LCL*	*UCL*	*p-value*
TGA	.	9.65	2.40	38.8	0.001
PC4	94.0%	0.85	0.76	0.96	0.01
DHEAS	59.0%	0.64	0.41	1.00	0.05
**Arrhythmia History**					
Variable	*Rel*	*OR*	*LCL*	*UCL*	*p-value*
TGA	.	5.96	1.54	23.1	0.01
Single ventricle	.	39.6	3.38	463	0.003
Age at assessment	.	1.06	1.01	1.10	0.01
PC2	97.2%	1.09	1.02	1.16	0.01
TMCA	100.0%	1.46	1.00	2.13	0.05
Orn/Arg	100.0%	1.22	1.05	1.43	0.01
**Arrhythmia History**					
Variable	*Rel*	*OR*	*LCL*	*UCL*	*p-value*
TGA	.	6.23	1.89	20.6	0.003
Single ventricle	.	20.1	1.61	251	0.02
Age at assessment	.	1.04	1.00	1.08	0.08
PC2	98.0%	1.08	1.01	1.15	0.02
TCA	99.8%	1.28	1.02	1.60	0.03
Orn/Arg	95.0%	1.19	1.01	1.41	0.03
**Shortness of Breath**					
Variable	*Rel*	*OR*	*LCL*	*UCL*	*p-value*
Ornithine	98.8%	1.05	1.02	1.08	<0.001
Homo-L-Arginine	95.8%	0.07	0.02	0.30	<0.001
SDMA/Arg	80.4%	1.15	1.03	1.29	0.01
**Exertional SOB**					
Variable	*Rel*	*OR*	*LCL*	*UCL*	*p-value*
Hypertension	.	2.64	1.15	6.05	0.02
PC1	89.6%	0.96	0.93	0.98	0.003
PC4	94.8%	0.90	0.82	0.98	0.02

Abbreviations: ADMA—asymmetric dimethylarginine; Arg—arginine; DHEAS—dehydroepiandrosterone sulfate; GCA—glycoholic acid; LCL—lower confidence limit; mets—metabolites; O_2—oxygen; OR—odds ratio; Orn—ornithine; PC—principal component; Rel—reliability in bootstrap resampling analysis; RV—right ventricular; SDMA—symmetric

dimethylarginine; SOB—shortness of breath; TGA—transposition of the great arteries; TMCA–tauromuricholic acid; UCL—upper confidence limit.

Table S2. Patient clinical status at the time of sample collection (number of patients = 99; number of assessments = 167) *.

	N	Septal Defects	N	Single Ventricle	N	TGA	N	TOF/RVOT	N	Other
Age (years)	33	43 ± 15	16	33 ± 8	45	37 ± 13	54	38 ± 14	19	33 ± 10
Systolic pressure (mmHg)	33	114 ± 17	16	117 ± 10	44	115 ± 18	54	127±17	19	123 ± 19
Heart rate (bpm)	33	74 ± 14	16	76 ± 11	43	72 ± 11	54	76 ± 14	19	77 ± 13
O2 saturation < 95%	33	5 (15%)	16	12 (75%)	44	2 (5%)	54	2 (4%)	19	2 (11%)
Current arrythmia	33	7 (21%)	16	13 (81%)	45	30 (67%)	54	7 (13%)	19	2 (11%)
Any cardiac dysfunction	15	7 (47%)	8	2 (25%)	19	12 (63%)	36	18 (50%)	12	0 (0%)
Any RV dysfunction	15	4 (27%)	6	2 (33%)	19	11 (58%)	35	16 (46%)	12	0 (0%)
RV volume overload	15	0 (0%)	5	0 (0%)	19	0 (0%)	35	13 (37%)	11	0 (0%)
Any LV dysfunction	15	6 (40%)	8	0 (0%)	18	2 (11%)	36	3 (8%)	12	0 (0%)
Shortness of breath	31	3 (10%)	16	5 (31%)	43	9 (21%)	51	10 (20%)	18	4 (22%)
Exertional SOB	32	10 (31%)	16	8 (50%)	43	20 (47%)	52	14 (27%)	18	3 (17%)
Anxiety/depression score	32	88 ± 13	16	71 ± 18	44	69 ± 24	53	83 ± 17	18	82 ± 16
Arrhythmia score	31	83 ± 17	16	68 ± 34	44	76 ± 26	52	82 ± 17	18	73 ± 20
Physical limitations score	32	64 ± 22	16	52 ± 25	44	57 ± 30	52	67 ± 24	18	68 ± 24
Quality of life score	32	76 ± 20	16	56 ± 20	44	63 ± 27	53	71 ± 23	18	74 ± 18
Symptoms score	32	80 ± 17	16	62 ± 26	44	69 ± 22	53	77 ± 18	18	74 ± 22
Total score	32	80 ± 15	16	63 ± 20	44	66 ± 23	53	77 ± 17	18	76 ± 17
Worsening quality of life	23	2 (9%)	14	1 (7%)	36	11 (31%)	39	4 (10%)	12	1 (8%)

N = Number of Assessments; RV volume overload—moderate or greater mitral or pulmonary regurgitation. Abbreviations: O2—oxygen; LV—left ventricular; RV—right ventricular. *Certain patients had more than one assessment during the study period.

Table S3. Non-cardiac diagnoses. Each space includes all non-cardiac diagnoses for each of the 71 patients in whom a non-cardiac diagnosis was present.

Types of Non-Cardiac Diagnoses
Patellar instability; Hypothyroid; Zoster; Hypercholesterolemia
Asthma
Obesity; Stenosis of R subclavian
Exercise induced asthma
Focal segmental glomerulosclerosis; Joint hypermobility; Recurrent pneumonias; Guillain barre history; Scoliosis
Hx of pancreatitis; Deep vein thrombosis; Seizure disorder; Stroke; Restrictive ventillatory defect; Hypothyroidism; Ashtma; Depression
Leukemia; Obstructive sleep apnea; Obesity; Down's Syndrome
Radial-ulnar synostoses
Anxiety; Attention deficit disorder; Large facial teratoma; Dysarthria likely due to deficient palate; Disequlibrium; Developmenntal delay; Menorrhagia
Psoriasis; Psoriatic arthritis; Essential thrombocytosis; Asthma; Obstructive sleep apnea; Obesity
Hypercholesterolemia
Pulmonary hypertension
Hypertension; Vascular disease involving the celiac axis and carotid arteries; Pulmonary hypertension
Asthma; Migraine; Fibromyalgia; Irritable Bowel Syndrome; Hepatitis C; Hypothyroidism
22q11 deletion syndrome; Acne vulgaris; Gingival hyperplasia
Hypertriglyceridemia; Allergic rhinitis
Cataracts; Depression; Cardiac cirrhosis; Obstructive sleep apnea
Raynauds; Seasonal allergies; Back pain
Paralyzed vocal cord; Alcoholism; Anxiety/depression; Obstructive sleep apnea; Chronically decreased vision in the left eye; Status post surgery to remove a supernumerary digit from his left thumb in infancy
Allegic rhinitis; Diverticulosis coli; Anxiety; Panic disorder; Pulmonary hypertension; Obesity; Snoring without sleep apnea; Non-alcoholic steatohepatitis; Thyroid nodules; Osteoarthritis

Obesity; Post traumatic stress disorder; Anxiety
Chronic sinusitis; Obesity; Hypothyroidism
Headaches; Endometriosis
Hyperlipidemia; Acne; Depression/anxiety; MTHFR gene mutation; Anemia; Lipomas
Carpal tunnel syndrome on left; Hepatitis C; Lymphadenopathy
Cohen's syndrome; Hypothyroidism; Seizures; Obesity
Pectus excavatum; Scoliosis; Obesity; Synostosis (cranial); Amenorrhea
Chronic osteoarthritis
Anemia; Depression; Gastroesophageal reflux disease; Hiatal hernia; History of kidney stones; Hyperlipidemia; Obesity; Obstructive sleep apnea
Obesity; Hyperlipidemia
Mild persistent asthma
Plantar fascial fibromatois;Osteoporosis
Hyperlipidemia
Gastroesophageal reflux disease; Gout; Iron deficiency; Obstructive sleep apnea; Pulmonary embolism; Transient ischemic attack
Rheumatoid arthritis
Gastroesophageal reflux disease; Psychotic affective disorder; Developmental delay; Sleep apnea; Hyperlipidemia; Obesity
Attention-deficit disorder; Down's syndrome; Hypothyroidism; Obesity; Obstructive sleep apnea; Pulmonary hypertension; Refractive amblyopia; Thyrotoxicosis history
Depression; Pulmonary hypertension
Nephrolithiasis
Hyperlipidemia; Thyroid disease; Migraine aura without headache
Obesity; Depression
Tracheal stenosis
Attention deficit disorder; Acne vulgaris
Obstructive sleep apnea
Sjogren's syndrome; Parenchymal lung disease; Migraine headace; Iron deficiency nemia; Gastroesophgeal reflux disease; Mixed connective tissue disease; Pectus excavatum; Scoliosis
Fracture of shaft of clavicle
Anxiety; Asthma; Down's syndrome; Eczema; Hypothyroidism; Obstructive sleep apnea
C7 spinal cord injury; Calculus of kidney; Chronic cystitis; Edema; Endometriosis; Hyperlipidemia; Osteoporosis; Pressure ulcer; Restrictive lung disease; Pressure sore on head
Congenital coxa vara; Pectus excavatum; Anxiety disorder; Dorsalgia
Hearing impairment; Cyanosis; Mental disability;
Interstitial lung disease; Pulmonary hypertension; Obstructive sleep apnea; Pulmonary tuberculosis; Hyperlipidemia
Obstructive sleep apnea; Epilepsy; Gastroesophageal reflux disease; Anoxic brain injury; Dysphagia; Psoriasis; Pulmonary hypertension; Seizures
Malignant neoplasm of prostate
Hyperlipidemia
Benign prostatic hyperplasia; Gastroesophageal reflux disease; Hernia; Hyperlipidemia; Hypertension; Nonalcoholic steatohepatitis; Chronic obstructive lung disease
Pulmonary hypertension
Anemia; Asthma; Congenital malrotation of intestine; Dysplasia of vocal cord; Dyspnea; History of omphalocele; History of small bowel obstruction; Nontoxic single thyroid nodule
Anxiety; Hyperlipidemia
Anxiety; Gout; Hypertrophy tonsils; Obesity
Attention deficit disorder; Asthma; Chronic depression; Hypersomnia; Nicotine dependence; Obesity
Anxiety; Fibroid; Gastroesophageal reflux disease; Hiatal hernia; viral pneumonia; Hypothyroidism

Asthma; Depression; Endometriosis; Hepatitis C; Hypercholesteremia; Migraine; Spondylosis, lumbar region; Intervertebral disc degeneration, lumbar region; Insomnia; Gastroesophageal reflux disease without esophagitis; Acute stress reaction
Hypothyroidism
Blindness; Brain cyst; Chronic obstructive pulmonary disease; Gastroesophageal reflux disease; Head trauma in child Migraines; Seizures
Thyroid disease; Cerebral vascular accident; Obstructive sleep apnea; Hyperlipidemia; Lung disease; Cervical cancer
Chronic kidney disease; Gout; Liver fibrosis; Obesity; Obstructive sleep apnea; Thyroid disease; Transient ischemi attack
Anxiety; Asthma; Thrombophlebitis
Asthma; Obesity; Obstructive sleep apnea
Malignant neoplasm of uterus
Anemia, iron deficiency; Breast cancer; Dysthymic disorder; Gastroesophageal reflux disease; Obstructive sleep apnea; Tracheal stenosis; Vasodepressor syncope; and Vocal cord paralysis; Hyperlipidemia
Anemia; Anxiety; Migraines; Spastic hemiparesis of left dominant side; Gastroesophageal reflux disease

Table S4. Significantly impacted pathways stratified by diagnoses (** FDR 0.01 < p < 0.05, * FDR 0.05 < p < 0.10, no * FDR > 0.10).

**Significantly Impacted Pathways**	
**Any cardiac dysfunction**	**Arrhythmia**
Arginine biosynthesis *	Valine, leucine and isoleucine biosynthesis*
Histidine metabolism *	Glutathione metabolism*
Butanoate metabolism *	Arginine biosynthesis
D-Glutamine and D-glutamate metabolism	Primary bile acid biosynthesis
Glutathione metabolism	Aminoacyl-tRNA biosynthesis
Alanine, aspartate and glutamate metabolism	Taurine and hypotaurine metabolism
Cysteine and methionine metabolism	Arginine and proline metabolism
Arginine and proline metabolism	
Aminoacyl-tRNA biosynthesis	**Worsened QOL**
	Aminoacyl-tRNA biosynthesis
**RV dysfunction**	Biosynthesis of unsaturated fatty acids
Arginine biosynthesis *	Histidine metabolism
Butanoate metabolism *	Sphingolipid metabolism
Histidine metabolism *	
D-Glutamine and D-glutamate metabolism *	
Glutathione metabolism	
Alanine, aspartate and glutamate metabolism	
Glycine, serine and threonine metabolism	
Cysteine and methionine metabolism	
Arginine and proline metabolism	
Aminoacyl-tRNA biosynthesis	
Sphingolipid metabolism	
**Shortness of breath**	
Glycine, serine and threonine metabolism	
Glycerophospholipid metabolism	
Arginine biosynthesis	
Histidine metabolism	
Citrate cycle (TCA cycle)	
**Exertional shortness of breath**	
Valine, leucine and isoleucine biosynthesis **	
Butanoate metabolism *	
D-Glutamine and D-glutamate metabolism	

Aminoacyl-tRNA biosynthesis
Alanine, aspartate and glutamate metabolism
Valine, leucine and isoleucine degradation
Citrate cycle (TCA cycle)
**Low oxygen saturation**
Primary bile acid biosynthesis **
D-Arginine and D-ornithine metabolism
Arginine and proline metabolism
Taurine and hypotaurine metabolism
Arginine biosynthesis
Butanoate metabolism

Abbreviations: FDR—false discovery rate; TCA—tricarboxylic acid cycle.

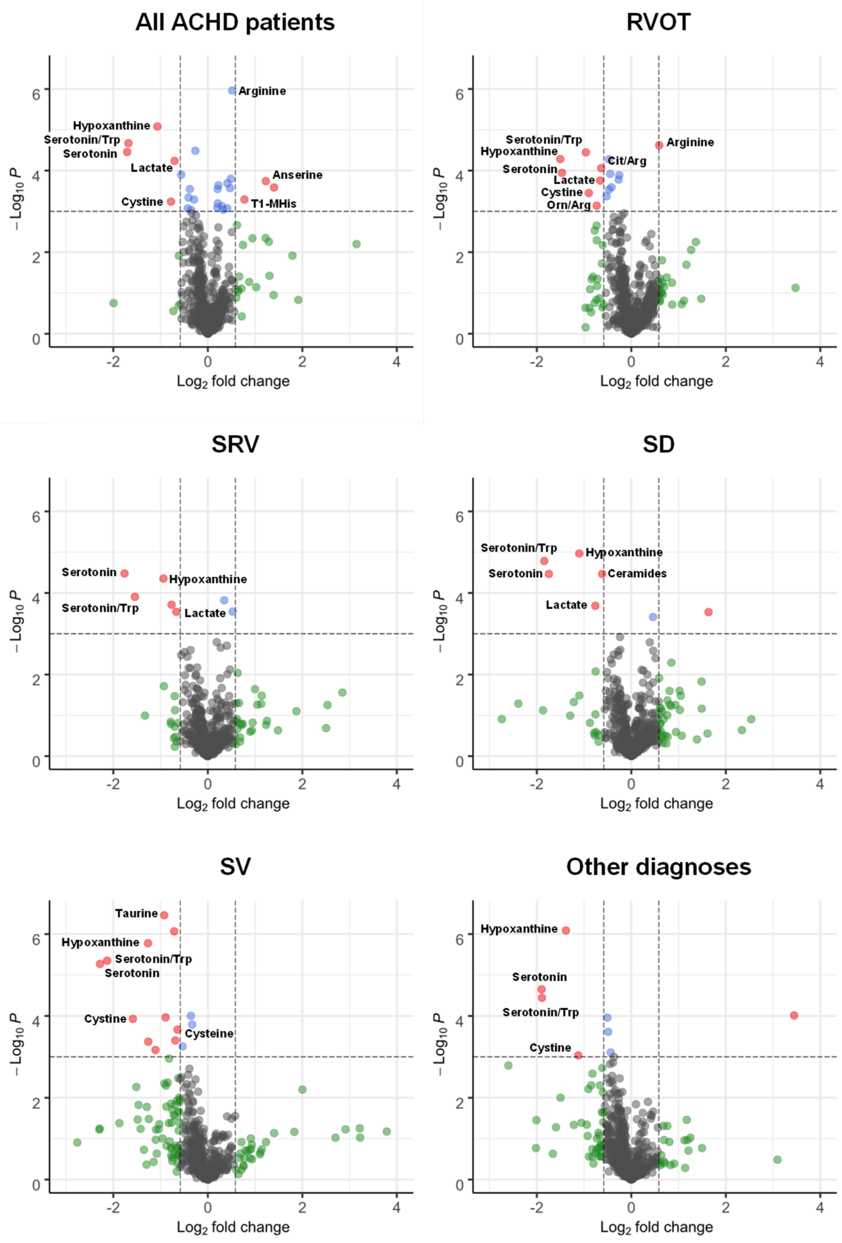

.

Figure S1. Volcano plots for differential expression of metabolites between controls and patients with ACHD, stratified by type of diagnosis (fold difference cutoff: 0.67/1.50, *p*-value cutoff: 0.001). Note:

Unlabelled red circles represent generic lipid-derived metabolites. Abbreviations: Arg—arginine; Cit—citrulline; Orn—ornithine; RVOT—right ventricular outflow stenosis; T1-MHis—1-Methylhistidine; TGA—transposition of the great arteries; TOF—Tetralogy of Fallot; Trp—tryptophan.

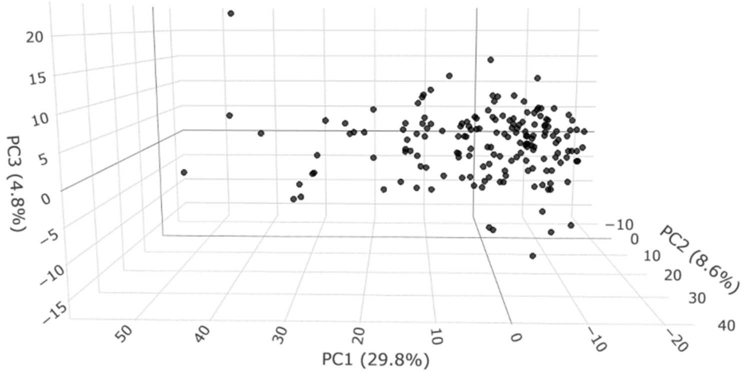

Figure S2. Principal cluster analysis plot in 3 dimensions.

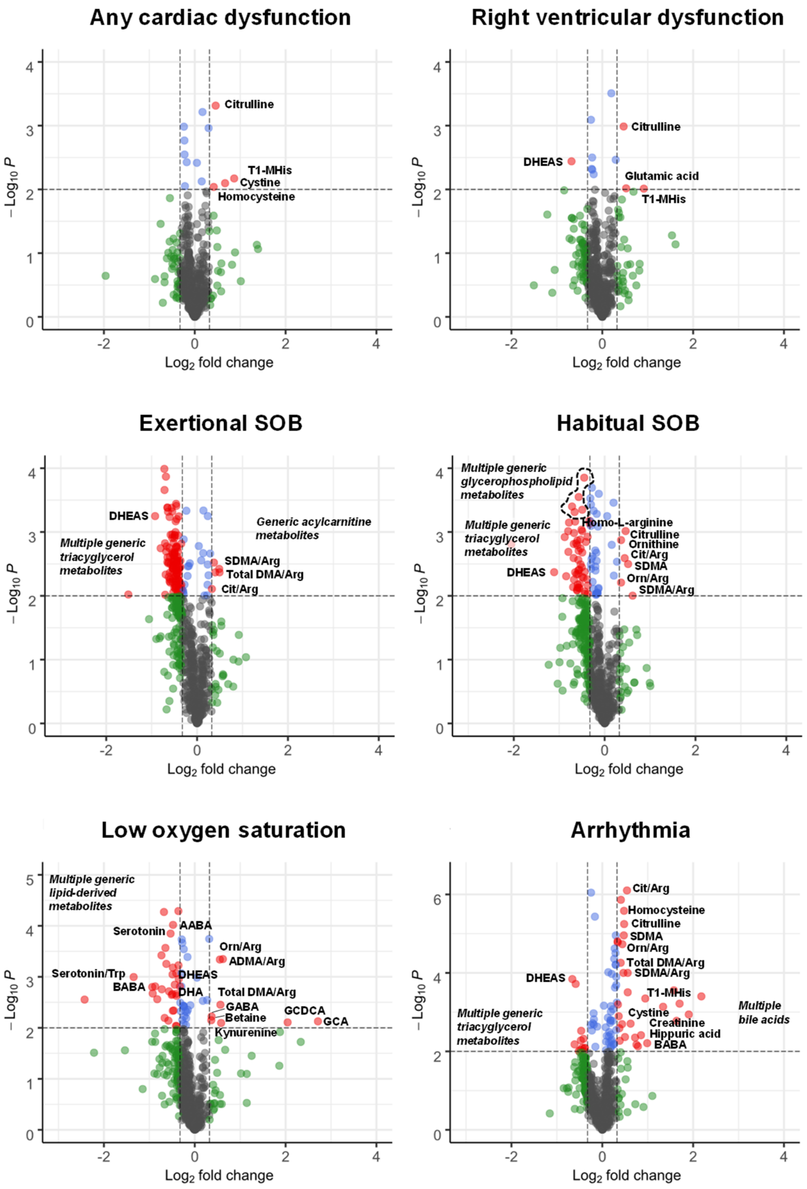

Figure S3. Volcano plots for differential expression of metabolites between patients with and without various clinical characteristics and outcomes (fold difference cutoff: 0.80/1.25, *p*-value cutoff: 0.01). Notes: For each graphs/areas, label in italic applies to all red circles that are unlabelled. In the habitual SOB panel, metabolites circles in dotted black line represent the generic glycerophospholipids metabolites, all other non-labelled metabolites in this area are generic triaglycerol metabolites. Habitual SOB—SOB not associated with exertion; Arrhythmia—history of arrhythmia. Abbreviations: ADMA—asymmetric dimethylarginine; ABBA—3-aminobutanoic acid; BABA—L-alpha-aminobutyric acid; Arg—arginine; Cit—citrulline; DHA—dosahexaenoic acid; DHEAS—dehydroepiandrosterone sulfate; DMA—dimethylarginine; GABA—gamma aminobutyric acid; GCA—glycocholic acid; GCDCA—chenodeoxycholic acid glycine conjugate; Orn—ornithine; SDMA—symmetric dimethylarginine; SOB—shortness of breath;T1-MHis—1-Methylhistidine; Trp—tryptophan.
